# Coexistence of Multiple Myositis-Specific Antibodies in Patients with Idiopathic Inflammatory Myopathies

**DOI:** 10.3390/jcm11236972

**Published:** 2022-11-25

**Authors:** Hung-Ling Huang, Wen-Chih Lin, Wei-Lun Tsai, Chia-Tse Weng, Meng-Yu Weng, Chun-Hsin Wu, Yuan-Ting Sun

**Affiliations:** 1 Department of Neurology, Tainan Hospital, Ministry of Health and Welfare, Tainan 700, Taiwan; 2 Department of Physical Medicine and Rehabilitation, Chiali Branch, Chi Mei Medical Centre, Tainan 722, Taiwan; 3 Department of Medical Genomics, National Cheng Kung University Hospital, College of Medicine, National Cheng Kung University, Tainan 701, Taiwan; 4 Department of Internal Medicine, Division of Allergy, Immunology, and Rheumatology, National Cheng Kung University Hospital, College of Medicine, National Cheng Kung University, Tainan 701, Taiwan; 5 Department of Neurology, National Cheng Kung University Hospital, College of Medicine, National Cheng Kung University, Tainan 701, Taiwan

**Keywords:** idiopathic inflammatory myopathies, myositis specific antibodies, exclusivity

## Abstract

The mutual exclusivity of myositis-specific antibodies (MSAs) has been reported before, but the coexistence of 2 or more MSAs was still found in a few case reports. This study aims to confirm the existence and prevalence of double MSAs in patients with idiopathic inflammatory myopathy (IIM) and to clarify the clinical features of these patients. One hundred fifty-one patients with IIM diagnosed from 1 July 2018 to 31 July 2022, at National Cheng Kung University Hospital, Taiwan, were enrolled and divided into two groups, patients with ≤1 MSA (*n* = 128, 84.8%) and those with ≥2 MSAs (*n* = 23, 15.2%) according to the initial serology results. After being re-examined by ANA-IIF assay, 8 out of 23 patients were confirmed to have ≥2 MSAs. The demographic data and clinical features were presented. The prevalence of double-positive MSAs among IIM was 5.3% in this cohort. The coexistence of two MSAs in an IIM patient does exist but is rare. Patients with two MSAs belonging to two distinct IIM subtypes presented clinical features skewed to one subtype instead of “mixed phenotypes”. No apparent difference in clinical severity was found between patients with ≥2 MSAs and ≤1 MSA. Longer follow-ups and more studies are required to characterize the patients of IIM with ≥2 MSAs.

## 1. Introduction

Idiopathic inflammatory myopathies (IIMs), collectively called myositis, are heterogeneous disorders characterized by muscle weakness, myalgia, elevated creatine kinase (CK) levels, and multiple extra-muscular manifestations, including variable degrees of skin, lung, or joint involvements. The revised classification of IIMs based on histopathology and myositis autoantibodies comprise dermatomyositis (DM), polymyositis (PM), inclusion body myositis (IBM), immune-mediated necrotizing myopathy (IMNM), and overlap myositis (OM) [1,2,3,4]. The subtyping of IIM was necessary because it was directly linked to the incidence of extra-muscular organ involvement, such as interstitial lung disease (ILD) [5,6] and concomitant malignancy [7].

Recent advances and the growing number of myositis autoantibodies have facilitated the diagnosis and classification of IIM [8,9,10,11,12]. Myositis autoantibodies can be divided into myositis-specific antibodies (MSAs) and myositis-associated antibodies (MAAs). MSAs are present only with specific subtype of IIM, whereas MAAs are not limited to IIM and are also found in other autoimmune diseases. The mutual exclusivity of MSAs has been reported before [13], which made the serology tests for MSAs more crucial for IIM subtyping. However, the coexistence of 2 or more MSAs was still found in a few case reports [14,15,16,17,18,19,20,21,22,23].

This study aimed to confirm the existence and the prevalence of double MSAs in patients with IIM and to clarify the clinical features of these patients.

## 2. Materials

### 2.1. Ethical Approval

Clinical studies were conducted according to the principles of the Nuremberg Code and the Declaration of Helsinki. The Institutional Review Board (IRB) of National Cheng Kung University Hospital approved the protocol (Approval No. B-BR-109-254). Because of the retrospective nature of the study, the IRB waived the requirement for informed patient consent.

### 2.2. Study Design and Subjects

**Figure 1:**
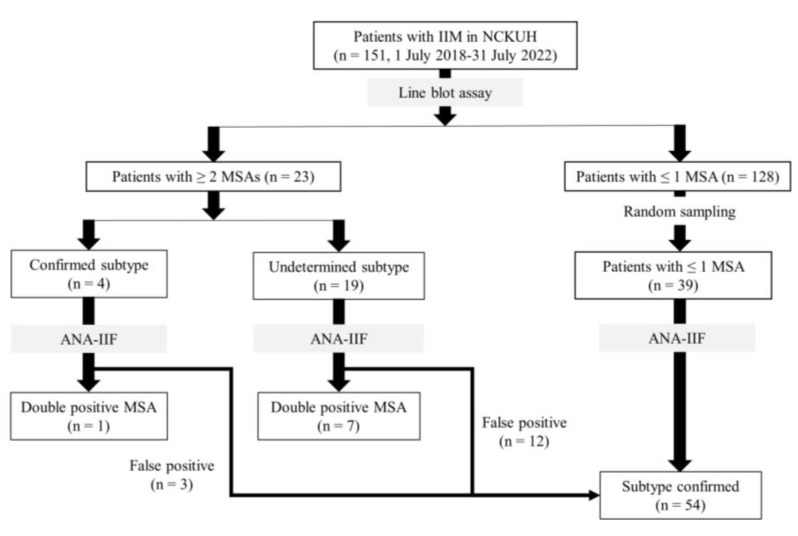
Flowchart of patient selection. IIM: idiopathic inflammatory myopathy; NCKUH: National Cheng-Kung University Hospital; MSA: myositis specific antibody; ANA-IIF: antinuclear antibody indirect immunofluorescence.

This retrospective case–control study was nested with a one-group pretest-posttest design. Patients with IIM diagnosed from 1 July 2018 to 31 July 2022, in National Cheng Kung University Hospital, Tainan, Taiwan, were enrolled and divided into two groups, patients with ≤1 MSA and those with ≥2 MSAs, according to the serology test results obtained from the line blot assay. The diagnosis of IIM was based on the EULAR/ACR Classification Criteria [24,25] and autoantibody serology. The one-group pretest-posttest design was nested in both groups of ≥2 MSAs and ≤1 MSA. To minimize the assay-related false positivities, samples with ≥2 MSAs shown by line blot tests were further tested by cell-based antinuclear antibody indirect immunofluorescence (ANA-IIF) assay [8,11]. Enzyme-linked immunosorbent assay (ELISA) for antibodies against melanoma differentiation-associated gene 5 (anti-MDA5) was applied for samples with the anti-MDA5 antibody on the line blot test. In parallel, in the group of ≤1 MSA, 39 serum samples were randomly picked out for ANA-IIF to confirm the result of the line blot test (Figure 1).

### 2.3. Demographic Data Collection

Clinical data were collected retrospectively through a comprehensive chart review. Muscle power was scored by the attending physician based on the medical research council (MRC) scale. Skin manifestations, such as heliotrope, Gottron’s sign, Gottron’s papule, V-sign, and shawl sign, were recorded. The CK level was documented when the patient presented to the clinic due to muscle weakness or relevant extra-muscular symptoms. Clinical course, comorbidities, treatments, periods of hospital stay due to flare-up of myositis, respiratory failure or not, reasons and date of mortality were all recorded.

### 2.4. Tests for Myositis Autoantibodies

#### 2.4.1. The Line Blot Assay

The line blot assay was carried out according to the instruction manual. In brief, fresh serum samples diluted in 100× were incubated with the pre-treated membrane-fixed immuno-blot for 30 min, in which the following MSAs are included, anti-Mi-2α, anti-Mi-2β, anti-TIF-1γ, anti-MDA5, anti-NXP-2, anti-SAE, anti-Jo-1, anti-SRP, anti-PL-7, anti-PL-12, anti-EJ, anti-OJ; and MAAs: anti-Ku, anti-PM-Scl100, anti-PM-Scl75, and anti-Ro52 (EUROIMMUN AG, Lübeck, Germany). After washing three times, the blot was incubated with enzyme conjugate for 30 min. Then, the blot was washed and incubated with a substrate for 5 min. The reaction was terminated by washing with distilled water. Afterward, dry the blot at room temperature. The intensity of bands was semi-quantified by the software EuroLineScan (Euroimmun AG, Lübeck, Germany) and graded as negative (−), equivocal (+/−), and the intensity of positivity was graded in tertile as +, ++, and +++ after comparing with positive and negative controls. In our cohort, graded as negative (−) or borderline (+/−) was considered a negative result.

#### 2.4.2. Cell-Based ANA Indirect Immunofluorescence

Residual serum thrown from −80 °C storage was tested with the Hep-2 cell-based IIF (EUROIMMUN AG, Lübeck, Germany), namely the ANA test. Diluted serum (40×) was incubated with fixed Hep-2 cells for 30 min, washed, and incubated with the fluorescent secondary antibody for another 30 min. Then, washed, mounted, visualized, and captured. The intensity of the images was quantified. The IIF pattern was confirmed by manual interpretation from 2 independent clinical laboratory technologists according to the international consensus on ANA patterns (ICAP) (Table 1) [8,10,26].

#### 2.4.3. Enzyme-Linked Immunosorbent Assay (ELISA)

Human interferon-induced helicase C domain-containing protein 1 ELISA Kit (FineTest, Wuhan, China) was used to quantify the level of anti-MDA5 antibody in human serum according to the instruction manual. In brief, the ELISA plate was washed twice, and then 100 μL standards and samples were added to each well. The plate was sealed with a cover and incubated for 90 min at 37 °C. After washing with wash buffer, a 100 μL biotin-labeled antibody working solution was added and incubated for 60 min at 37 °C. Then, 100 μL HPR-Streptavidin conjugate (SABC) working solution was added to each well and incubated for 30 min at 37 °C after washing. Finally, the wells were washed with wash buffer five times, keeping the buffer in the well for 2–3 min each time, then 90 μL TMB substrate was added to each well. The plate was incubated at 37 °C in the dark for 10–20 min. Afterward, 50 μL stop solution was added immediately and read at O.D. 450 nm.

## 3. Results

From July 2018 to July 2022, 151 patients with IIM (59.97 ± 14.33 years old, 66.2% female) at National Cheng Kung University Medical Center received a myositis autoantibody line blot test. Among them, 128 (84.8%) had ≤one MSA, and 23 (15.2%) had ≥two MSAs. When the ≥two coexisted MSAs belonged to the same subtype of IIM, the patient was allocated to the “confirmed subtype” undoubtedly. In contrast, while the ≥ two coexisted MSAs belonged to different subtypes of IIM, patients were assigned to the “subtype undetermined” group. Four of the 23 patients with ≥two MSAs had confirmed IIM subtypes, and 19 had undetermined IIM subtypes.

### 3.1. The Prevalence of Double-Positive MSAs in IIM

The serum samples of the 23 patients with ≥two MSAs in the first serology test were re-examined by cell-based ANA pattern assay. After reading through and reaching an agreement with two technologists, the concluded ANA pattern, the titer, and the representative immunofluorescence images were summarized in Table 2. Because MDA5 was not expressed well on Hep-2 cells, the ANA pattern of the anti-MDA5 antibody can be negative or cytoplasmic [10,27]. Thus, we used the ELISA assay to confirm the positivity of the anti-MDA5 antibody.

After comparing the ANA pattern with the line blot results, 3 out of 4 samples in the “confirmed subtype” group were finally proved that one of the coexisted MSA on line blot was a false positive. The true positive MSA was TIF-1γ in patient 1, MDA5 in patient 2, and patient 12 (Table 2). The false-positive antibodies were MDA5 in patient 1, SAE antibody in patients 2 and 12. Only one subject in the “confirmed subtype” group, patient 5, had truly double-positive MSAs: the coexistence of anti-MDA5 and anti-Mi-2β antibodies, Table 2).

In the “subtype undetermined” group (*n* = 19), six patients had coexistence of 2 MSAs suggested DM and IMNM, two patients had 2 MSAs suggested OM and IMNM, ten patients had 2 MSAs belonged to DM and OM, and one patient had 3 MSAs suggested DM, OM, and IMNM. Seven out of 19 patients in the “subtype undetermined” group were confirmed to have double-positive MSAs. The prevalence of double-positive MSAs among IIM was 5.3% (8/151). None had triple-positive MSAs.

### 3.2. The False-Positive Rate of Immunoassays

In the group with ≤1 positive MSA, the ANA patterns of the randomly picked up 39 samples were compared to the line blot assay results. Twenty-nine out of 39 patients have confirmed single positive MSAs: 6 had anti-Jo-1, 6 had anti-PL-7, 4 had anti-SRP, 3 had anti-MDA5, 2 had anti-EJ, 2 had anti-TIF-1γ, 2 had anti-PL-12, 1 had anti-SAE, 1 d had anti-NXP-2, 1 had anti-Mi-2β, and 1 had anti-Mi-2α antibody, while the rest were seronegative. Nearly all subjects (*n* = 38) had consistent results between the above two tests, except for one patient. This patient’s serum was tested positive for anti-OJ antibody via line blot assay at three different time points. However, his ANA-IIF suggested an unrecognizable antibody at the nuclear membrane with a titer of 1:640 and 1:1280. The clinical manifestations of this patient, including myalgia, arthritis, and fever, suggested a favorable diagnosis of antisynthetase syndrome or overlap myositis. Even though the MSA serology result from line blot was inconsistent with the ANA-IIF, the final diagnosis of this patient remained.

In the group with ≥2 MSAs, the false positive antibodies on line blot were summarized as follows: There were 2 false positive MDA5 (both graded as +), 3 SAE (all graded as +), 2 PL-7 (graded as +), 7 SRP (4 graded as +, 2 graded as ++, and 1 graded as +++), 1 Mi-2α (graded as +), 2 EJ (graded as ++ and +++, respectively), 3 Jo-1 (graded as +,+, and ++, respectively), 2 NXP-2 (graded as + and ++, respectively), 2 TIF-1γ (all graded as +), one OJ (graded as +) and one PL-12 (graded as +). Among all MSAs, the false positive rate was 100% (1/1) for PL-12, 70% (7/10) for SRP, 66.7% (2/3) for EJ, 66.7% (2/3) for NXP-2, 50% (3/6) for SAE, 50% (1/2) for Mi-2α, 50% (1/2) for OJ, 50% (2/4) for PL-7, 42.9% (3/7) for Jo-1, 33.3% (2/6) for MDA5, 28.6% (2/7) for TIF-1γ, and 0% (0/2) for Mi-2β.

### 3.3. The Clinical Features of IIM Patients with Double-Positive MSAs

The demographic and clinical data of the eight “true” double-MSA patients were summarized in Table 3. Female patients accounted for 75% (6/8). The average age of onset was 56.3 years old. The age and sex distribution were not different from the entire cohort of IIM. Two patients (Patient 5 and Patient 20 in Table 3) had two antibodies belonging to the same IIM subtype, DM, and also presented typical signs. The other five patients had two antibodies belonging to the distinct IIM subtypes. They showed typical signs predominant to one of the two IIM subtypes instead of mixed features of the two IIM subtypes. For instance, patients 19 and 23 had double positive anti-SRP and anti-SAE antibodies, but one developed typical clinical symptoms of DM, and the other presented as IMNM. Patients 17 and Patient 18 had coexisted anti-PL-7/anti-TIF-1γ and anti-Jo-1/anti-TIF-1γ antibodies, respectively. They were diagnosed as OM instead of DM according to their clinical manifestations and muscle pathology. The two patients had no malignancy found during the follow-up period, although the anti-TIF-1γ antibody was reported to be associated with a higher risk for cancer [28,29]. Patient 7, 17, and 18 had comorbidities of ILD. Patient 5 and patient 17 were found to have overlapping rheumatoid arthritis (RA). Overall, no respiratory failure or mortality happened during the follow-up period in all myositis patients with double MSA.

## 4. Discussion

This study demonstrated a 5.3% of double positive MSAs in patients with IIM after excluding false positivity by additional immunoassay. Their final diagnoses were DM (*n* = 5), OM (*n* = 2) and IMNM (*n* = 1). Patients with double MSAs had the following features: Firstly, the severity and extensiveness of clinical phenotypes in these double-MSA IIM patients were similar to IIM patients with single-positive MSAs. Secondly, while presenting two antibodies belonging to two distinct IIM subtypes, their phenotypes skewed to one of them. No “mixed phenotypes” from the two different IIM subtypes were found in our cohort. Thus, a muscle biopsy will be helpful when the clinical features and the coexisted MSAs belonging to the distinct IIM subtypes confuse the diagnosis [30]. Our results did not go against the notion that the presence of MSAs was essentially mutually exclusive in IIM [13]. The coexistence of two MSAs in an IIM patient is rare but does exist [14,15,16,17,18,19,20,21,22,23].

With the double confirmation of additional immune assays, we found a high false-positive rate (around 33.3% (28/84)) of the line blot test, particularly in samples with ≥2 MSAs (48.1%, 25/52). The high false positive rate in multiple positive blots suggested possible systematic issues that alter the cutoff between positive and negative values, such as human error or the quality control of the assay kit. Or it reflected the varied specificity of each antigen or autoantibody.

The accuracy of serology test was of clinical significance because of the developing serology-based model for disease prognosis [31,32]. Thus, when the coexisted MSAs belonged to separate IIM subtypes, a careful confirmation by ANA-IIF, ELISA, or immunoprecipitation was recommended for the precise subtyping of IIM. The IIM subtyping was crucial because it was relevant to the frequency of tumors and the coexisted extra-muscular phenotypes, such as ILD. In this study, the second assay identified two-thirds of patients with false-positive results.

Compared with IIM patients with ≤1 MSA, the age of patients with double MSA showed no significant difference in this cohort (≤1 MSA and double MSA groups, aged 52.3 and 56.3 years, respectively; *p* = 0.54). Due to the fact that DM is the most prevalent IIM subtype, we also compared the age of DM patients with double MSAs (*n* = 5, aged 54.2 years) to DM patients with single or zero MSA (*n* = 14, aged 57 years), and the result revealed no statistical difference (*p*-value = 0.762). The sex distribution was also similar (≤1 MSA and double MSAs groups, 74.4 and 75% female, respectively; *p* = 0.71). Compared with previous studies, Bodoki L. reported a 1.8% prevalence double positive MSAs in IIM patients by using immunoblot (Orgentec Diagnostika) and radiolabelled immunoprecipitation (RIA) [22], Betteridge Z. reported a 0.2% prevalence by using RIA, and ELISA, particularly for anti-MDA5 and anti-NXP-2 antibodies [13] (Table 4), the prevalence of double seropositivity in our cohort was slightly higher. Like previous studies, DM was the most common clinical diagnosis in IIM patients with ≥2 MSAs [14,17,18,21].

Because of the low prevalence of double seropositive IIM, the frequency of various combinations of MSAs remained unknown. In our eight double seropositive cases, except for the coexistence of anti-SRP and anti-Mi-2β antibodies (Patient 4), the other combinations had not been reported before: anti-MDA5 plus anti-Mi-2β (Patient 5), anti-Jo-1 plus anti-NXP-2 (Patient 7), anti-PL-7 plus anti-TIF-1γ (Patient 17), anti-SRP plus anti-SAE (Patient 19 and 23), anti-MDA5 plus anti-TIF-1γ antibodies (Patient 20), anti-Jo-1 plus anti-TIF-1γ (Patient 18). Whether there is a common rule for the combination of MSAs needs further studies.

Regarding the clinical features, patients with double MSAs in this cohort presented predominant features skewed to either IIM subtypes instead of mixed features corresponding to the coexisted two MSAs. For instance, our Patient 19 presented both anti-SRP and anti-SAE antibodies manifested as amyotrophic DM, without typically profound muscle weakness as anti-SRP-related IMNM. However, a few reports showed mixed features of two IIM subtypes in the double-seropositive cases. Malkan A. reported a case with the coexistence of anti-SRP and anti-PL-12 antibodies, presenting with “mixed features” of both IMNM and antisynthetase syndrome [21].

In addition, there seems to be no hierarchy in coexisted MSAs when presenting relevant clinical features. For incidence, in patients with anti-SRP and anti-Mi-2β antibodies, our Patient 1 manifested as amyotrophic DM, more like the pertinent presentations to anti- Mi-2β antibody. In contrast, the case reported by Akintayo RO presented profound muscle weakness without skin rash, more like the presentation of anti-SRP IMNM [20]. In the case series from Hungarian IIM patients, one patient with anti-SRP and anti-Mi2 manifested polymyositis without skin rash [22].

Regarding the severity of clinical presentations, Sugie K. stated that the coexistence of 2 MSAs, as in his case with anti-Jo-1 and anti-SRP antibodies, may lead to more severe clinical symptoms [23]. However, we did not find a more severe clinical course in double seropositivity patients in this cohort. Our cases had no respiratory failure or mortality during follow-up. Besides, patients 4, 5, and 19 manifested amyotrophic DM without apparent muscle weakness.

About the comorbidities, 3 out of 8 patients (37.5%) had ILD in our cohort. High frequency of concomitant ILD was noted in previous studies (summarized in Table 4). In total, 14 out of 18 cases (77.8%) with double MSAs listed in Table 4 had ILD. A previous report addressed a higher frequency (2 out of 12 patients) of overlapped RA in IIM patients with double MSAs [33]. Two subjects (Patient 5 and Patient 17) in our cohort also had RA. Patient 5, with anti-MDA5 and anti-Mi-2β antibodies presented DM first, followed by RA. Patient 17, with anti-PL-7 and anti-TIF-1γ antibodies had RA first and was subsequently diagnosed with OM. However, the clinical relevance between arthritis and double-seropositive IIM requires more studies.

In conclusion, the coexistence of ≥2 MSAs does exist but is rare. Additional immune assays are recommended to confirm the double positive results in the line blot assay and exclude false positive cases. Applying the widely used cell-based ANA pattern in this study is convenient and efficient [34]. Most patients with ≥2 MSAs clinically resembled DM, which is also one of the most commonly seen IIM. No precise rule was noted in the combination of 2 MSAs. We did not find an MSA that is persistently superior to another MSA regarding its relevance to clinical presentations. No mixed phenotype or more severe course was found in this cohort. Finally, further accumulation of such cases is necessary for clarifying the features of the coexistence of 2 MSAs and whether differences in profile patterns translate into varying clinical phenotypes.

## Figures and Tables

**Table 1:** Summary of IIF pattern and main features of MSAs.

MSA	IIF HEp-2 [10,26]	Clinical Association [10]
Anti-Jo-1	Cytoplasmic fine speckled	Classic antisynthetase syndrome with more frequent muscle involvement
Anti-PL-7	Cytoplasmic dense fine speckled	Anti-synthetase syndrome with prevalent ILD
Anti-PL-12	Cytoplasmic dense fine speckled	Anti-synthetase syndrome with prevalent ILD
Anti-EJ	Cytoplasmic speckled	Anti-synthetase syndrome
Anti-OJ	Cytoplasmic speckled	ILD alone or antisynthetase syndrome
Anti-Mi-2α, anti-Mi-2β	Fine speckled	Classical DM
Anti-MDA5	Negative, or cytoplasmic speckled	Hypo-amyopathic, ILD with possible RP-ILD and severe and peculiar skin involvement
Anti-SAE	Fine speckled	Severe cutaneous disease that classically precede DM with severe dysphagia and systemic symptoms
Anti-TIF-1γ	Fine speckled	Juvenile DM. Cancer-associated hypo-myopathic DM
Anti-NXP-2	Fine speckled or multiple nuclear dots	Juvenile DM, diffused calcinosis. Cancer-associated DM
Anti-SRP	Cytoplasmic dense fine speckled	IMNM with frequent esophageal involvement. Possible ILD

Abbreviations: MSA = myositis specific antibody; IIF = indirect immunofluorescence; ILD = interstitial lung disease; DM = dermatomyositis.

**Table 2:** Summary of ANA pattern of 23 patients with double seropositivity in line blot test.

Patient	ANA Pattern		Line Blot Assay	True Positive	False Positive
1	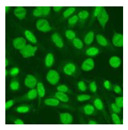	1: 160 AC4 Fine speckled 1: 80 AC8/AC9 nucleolar 1:40 AC19/AC20 Cytoplasmic speckled	MDA5 + TIF-1γ++	TIF-1γ	MDA5
2	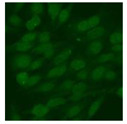	Negative	SAE+ MDA5+	MDA5	SAE
3	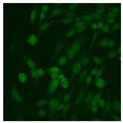	1:40 AC17 Segmental Fibrillar 1:80 AC-24 Centrosome	PL-7+ SRP++	-	PL-7 SRP
4 *	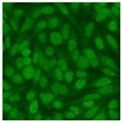	1: 40 AC4 Fine speckled 1:40 AC19/AC20 Cytoplasmic speckled	SRP+ Mi-2β++	SRP Mi-2β	-
5 *	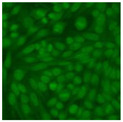	1: 40 AC3 Centromere 1:40 AC4 Fine speckled 1: 40 AC19/AC20 Cytoplasmic speckled	MDA5+++ Mi-2β+	MDA5 Mi-2β	-
6	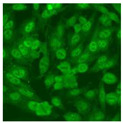	1:160 AC5 Large/coarse speckled 1:80 AC18/AC19/AC 20 Cytoplasmic speckled	Jo-1+++ Mi-2α+	Jo-1	Mi-2α
7 *	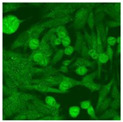	1: 320 AC6 Multiple nuclear dots 1:80 AC18/AC19/AC20 Cytoplasmic speckled	Jo-1+++ NXP-2+	Jo-1 NXP-2	-
8	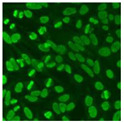	1:80 AC1 Homogenous/unclassfical nuclear	EJ++ PL-7+ Jo-1+ NXP-2+	-	EJ PL-7 Jo-1 NXP-2
9	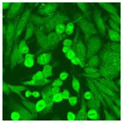	1:160 AC6 Multiple nuclear dots 1:160 AC20 Cytoplasmic Fine Speckled	EJ+++ Jo-1+++ TIF-1γ+	Jo-1	EJ, TIF-1γ
10	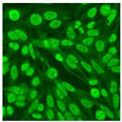	1: 320 AC4/AC5 Speckled 1:160 AC9 Clumpy nucleolar 1: 40 AC19 Cytoplasmic dense fine speckled	Jo-1+ TIF-1γ+++	TIF-1γ	Jo-1
11	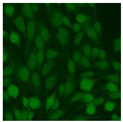	1: 40 AC4/AC5 Speckled 1:40 AC 27 Intercellular bridge	SRP+ SAE+	SAE	SRP
12	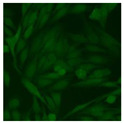	Negative	SAE+ MDA5+	MDA5	SAE
13	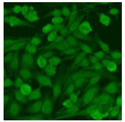	1:40 AC4 Fine speckled 1:40 AC19 Cytoplasmic dense fine Speckled	OJ+ Jo-1++ MDA5+	-	OJ Jo-1 MDA5
14	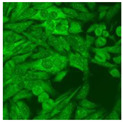	1:160 AC6 Multiple Nuclear dots 1:160 AC20 Cytoplasmic fine Speckled	EJ+++ SRP++	EJ	SRP
15	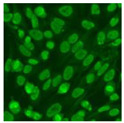	1:160 AC1 Homogeneous 1:160 AC4 Fine speckled	SRP+ Mi-2α+	Mi-2α	SRP
16	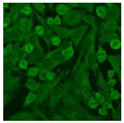	1:40 AC6 Multiple Nuclear dots 1:160 AC19/AC20 Cytoplasmic Speckled	PL-7+++ TIF-1γ+	PL-7	TIF-1γ
17 *	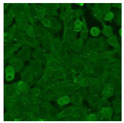	1:40 AC4 Fine speckled 1:40 AC7 Few nuclear dots 1:40 AC19/AC20 Cytoplasmic Speckled	PL-7+++ TIF-1γ++	PL-7 TIF-1γ	-
18 *	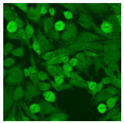	1: 80 AC4/AC5 Speckled 1: 80 AC19 Cytoplasmic dense fine speckled	Jo-1+++ TIF-1γ++	Jo-1 TIF-1γ	-
19 *	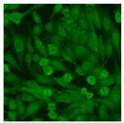	1:80 AC4 Fine speckled 1:40 AC19 Cytoplasmic dense fine Speckled 1:40 AC27 Anti-Midbody	SRP+ SAE+++	SRP SAE	-
20 *	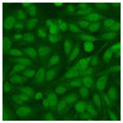	1:80 AC4 Fine speckled 1:40 AC19/AC20 Cytoplasmic Speckled 1:40 AC27 Anti-Midbody	PL-12+ SRP+ SAE+ MDA5+ TIF-1γ+++	MDA5 TIF-1γ	PL-12 SRP SAE
21	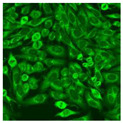	1:320 AC 19 Cytoplasmic dense Fine speckled 1:160 AC 22 Polar/Golgi-like	SRP+++ NXP-2++	SRP	NXP-2
22	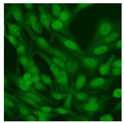	1:80 AC4/AC5 speckled 1:40 AC19/20 Cytoplasmic speckled 1:40 AC24 Centrosome	OJ++ SRP+	OJ	SRP
23 *	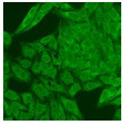	AC4—Nuclear Fine Speckled AC19—Cytoplasmic Dense Fine Speckled	SRP+++ SAE+	SRP SAE	-

* ≥2 MSAs via cell-based ANA pattern. Abbreviations: ANA = antinuclear antibody; AC = anti-cell.

**Table 3:** Summary of clinical features of 8 patients of IIM with double positive MSAs.

	Age */Gender	MSA	Clinical Diagnosis	Muscle Symptoms	Skin Manifestations	CK (u/l)	Pathology	Comorbidities	Treatment	Mortality
Patient 4	51F	SRP Mi-2β	Dermatomyositis	No weakness	heliotrope, V-sign, and Gottron’s sign	39	Skin biopsy: DM	-	Prednisolone, MTX	-
Patient 5	29F	MDA5 Mi-2β	Dermatomyositis	No weakness; soreness sometimes	Facial rash	38	-	RA	Prednisolone, MTX, hydroxychloroquine	-
Patient 7	63F	Jo-1 NXP-2	Dermatomyositis	Proximal limbs weakness	Heliotrope, Raynaud’s phenomenon	1685	Muscle biopsy: DM	ILD, secondary Sjogren syndrome	Prednisolone, AZA, hydroxychloroquine, sulfasalazine	Loss f/u since July 2020
Patient 17	53F	PL-7 TIF-1γ	Overlap myositis	Proximal limbs weakness	Mechanic’s hands	5053	Muscle biopsy: OM	RA, ILD, HL, diabetes mellitus	Prednisolone, AZA, sulfasalazine	-
Patient 18	67F	Jo-1, TIF-1γ	Overlap myositis	Proximal limbs weakness	-	574	-	ILD	Prednisolone, AZA	-
Patient 19	66F	SRP SAE	Dermatomyositis	No weakness	erythematous change over scalp, forehead, V-sign, shawl sign, Gottron’s sign	104	Skin biopsy: DM	-	Hydroxychloroquine	-
Patient 20	62M	MDA5 TIF-1γ	Dermatomyositis	Proximal limbs weakness	Gottron’s sign, V-sign	1209	Muscle biopsy: DM	-	Prednisolone, AZA, hydroxychloroquine	-
Patient 23	59M	SRP SAE	Immune-mediated necrotizing myopathy	Proximal limbs weakness	-	1773	Muscle biopsy: IMNM	HBV, HL	Prednisolone, MTX	

* Age: age of onset. Abbreviations: MSA = myositis specific antibody; CK = creatine kinase; DM = dermatomyositis; OM = overlap myositis; IMNM = immune-mediated necrotizing myopathy; ILD = interstitial lung disease; RA = rheumatoid arthritis; HL = hyperlipidemia; AZA = azathioprine; MTX = methotrexate.

**Table 4:** Literature review.

Study	Age/Gender	Diagnosis	Antibody	Test of Ab	Treatment	Comorbidities	Prognosis
Li ZY [14]	27F	Amyotrophic DM	MDA5, PL-7	Immunoblot for twice	Respiratory failure, s/p ECMO	RPILD, chronic hirsutism, oligomenorrhea	Expired
Ito M [15]	33F	Typical DM	Mi-2, NXP-2	IIF, ELISA, IP, IP-western assays	Oral prednisolone	-	Monophasic
Takeuchi Y [17]	53F	Amyotrophic DM	EJ, MDA5	IP, ELISA	Oral prednisolone, tacrolimus, cyclosporine, P/E	ILD	Polyphasic (ILD flare up 4+ times)
Huang L [18]	32M	Amyotrophic DM	HMGCR, MDA5	IIF, Immunoblot, IP	-	ILD	Expired
	39M	Amyotrophic DM	HMGCR, MDA5	IIF, Immunoblot, IP	-	ILD	Expired
	43F	Amyotrophic DM	HMGCR, MDA5	IIF, Immunoblot, IP	-	ILD	
	57F	PM	HMGCR, Jo-1	IIF, Immunoblot, IP	-	ILD	
	24M	Amyotrophic DM	HMGCR, MDA5	IIF, Immunoblot, IP	-	ILD	
Naniwa T [19]	43F	Classic DM	MDA5, PL-7	IP, ELISA, immunoblot	High dose steroid, IV cyclophosphamide, IVIG, tacrolimus, AZA	RPILD	Polyphasic
Akintayo RO [20]	19M	Profound weakness, no rash	SRP, Mi-2	IP	High dose steroid, AZA, mycophenolate	-	Monophasic
Malkan A [21]	37F	IMNM, antisynthetase syndrome	SRP, PL-12	Not mentioned	High dose steroid, IVIG, AZA, IV cyclophosphomide	ILD	chronic
Bodoki L [22]	70F	PM	Jo-1, SRP	Immunoblot, IP	Steroid, cyclophosphomide	ILD	chronic
	49F	DM	Jo-1, SRP	Immunoblot, IP	Steroid, cyclophosphomide	ILD	Monophasic
	50F	PM	Jo-1, Mi-2	Immunoblot, IP	Steroid, AZA	ILD, HTN, obesity	Polyphasic
	68M	DM	Mi-2, PL-12	Immunoblot, IP	Steroid	ILD	Monophasic
	37F	PM	Mi-2, SRP	Immunoblot, IP	Steroid, cyclophosphamide, IVIG, cyclosporine, methotrexate	-	Chronic
	43M	PM	SRP, PL-7	Immunoblot, IP	Steroid, methotrexate	-	Polyphasic
Sugie K [23]	61M	DM	Jo-1, SRP	IIF	Oral steroid	ILD, gastric cancer	Expired

Abbreviations: Ab = antibody; DM = dermatomyositis; RPILD: rapid progressive interstitial lung disease; ECMO: Extracorporeal membrane oxygenation; IIF: indirect immunofluorescence; ELISA: Enzyme-linked immunosorbent assay; IP: immunoprecipitation; ILD: interstitial lung disease; P/E: plasmapheresis; PM: polymyositis; AZA = azathioprine; IVIG: Intravenous immunoglobulin.

## Data Availability

The data are available upon request from the corresponding author.
